# Harsh Parenting and Children’s Aggressive Behavior: A Moderated Mediation Model

**DOI:** 10.3390/ijerph19042403

**Published:** 2022-02-19

**Authors:** Bowen Liu, Yuhua Yang, Jie Geng, Tingting Cai, Mengjuan Zhu, Tao Chen, Jinjing Xiang

**Affiliations:** School of Humanities and Social Sciences, Beijing Forestry University, Beijing 100083, China; liubowen0622@163.com (B.L.); yangyuhua617@163.com (Y.Y.); gjimut@163.com (J.G.); ctt20220123@126.com (T.C.); z1072492615@163.com (M.Z.); chewns@163.com (T.C.)

**Keywords:** harsh parenting, aggressive behavior, normative beliefs about aggression, regulatory emotional self-efficacy, integrated model of emotion processes and cognition

## Abstract

Harsh parenting and its effect on children’s aggressive behavior has received attention from researchers, however few studies have considered the role of the emotional process. This study aims to examine the relationship between harsh parenting, children’s aggressive behavior, normative beliefs about aggression, and regulatory emotional self-efficacy, alongside their mechanism of interplay. A sample of 235 senior primary school students in Beijing were recruited as participants by using the Harsh Parenting Scale, the Normative Beliefs about Aggression Scale, the Buss–Warren Aggression Questionnaire, and the Regulatory Emotional Self-Efficacy Scale. Results indicated that: (1) Harsh parenting had a significant positive predictive effect on children’s aggressive behavior after controlling gender; (2) normative beliefs about the aggression of children mediated the relationship between harsh parenting and children’s aggressive behavior; and (3) regulatory emotional self-efficacy had moderating effects both the mediation model of normative beliefs about the aggression of children and in the direct predictive model of harsh parenting on children’s aggressive behavior. The results are not only helpful to understand the relationship between harsh parenting and children’s aggressive behavior from the perspective of an integrated model of emotion processes and cognition, but also provide a new practical way to prevent and intervene in children’s aggressive behavior in the future.

## 1. Introduction

Aggressive behavior refers to disruptive behavior that intentionally causes physical or psychological harm to others [1]. Aggressive behavior can be divided into three forms: physical aggression, verbal aggression, and relational aggression [2]. Children’s aggressive behavior can trigger many negative consequences. On the one hand, relatively high aggressiveness and obvious aggressive behavior in children may affect the development of their attention, reduce academic performance, and produce negative emotions [3,4,5]. On the other hand, aggressive behavior is closely related to peer victimization and bullying [6]. People involved in bullying may have more internalizing problems, and even develop suicidal ideation [7], resulting in more serious consequences. While aggression can continue to develop and have a lasting impact in adolescence and adulthood [8], externalized behavioral problems generally tend to be stable from early childhood [9]. Therefore, it is necessary to further understand the development mechanism of children’s aggressive behavior and the factors affecting its development, so as to provide a new direction for the practical work of related prevention and intervention measures.

Parenting style is an important factor affecting children’s physical and mental development. Harsh parenting involves a wide range of negative parental styles towards children such as violent behavior, verbal aggression, and savage manners, which have negative effects on children [10]. According to the social learning theory, parents’ rude attitudes or behaviors towards themselves or others will gradually be acquired through observation and imitation by children in the early growth due to their lacking ability in rational judgment and other references, and then internalized into their own attitudes and behaviors in dealing with problems [11]. Empirical study also has demonstrated that individuals exposed to harsh parenting have low empathy, manipulative desire, negative self-awareness, and hostile attitude towards society and others. Their parent–child relationship and interpersonal skills are generally worse, and behavioral problems such as traditional aggression and relationship aggression are more likely to occur [12,13,14]. Previous studies on harsh parenting and children’s aggressive behavior were mostly discussed from an emotional level or cognitive level, respectively [15,16]. Therefore, more process-oriented research is needed. This study will consider children’s emotional and cognitive factors comprehensively to further explore the relationship between harsh parenting and children’s aggressive behavior.

### 1.1. The Mediating Role of Normative Beliefs about Aggression

Normative beliefs about aggression refers to the extent to which individuals think aggression is a reasonable and acceptable social behavior. It can be divided into general normative beliefs and situational normative beliefs. General normative beliefs about aggression holds that “Generally speaking, it is OK to beat people. It’s my right”. While situational normative beliefs about aggression holds that “If someone hit me first, I will fight back” [17]. Parenting style can directly or indirectly affect their children’s internalization of social behavior norms [18]. The general aggression model also points out that long-term exposure to a violent environment will improve an individual’s aggression cognition, and regard aggressive behavior as a reasonable means to solve conflicts, thus establishing high normative beliefs about aggression and make it easier to produce aggressive behavior [19]. In brief, harsh parenting does not demonstrate reasonable emotional and behavioral control strategies to individuals. It can lead to emotional disorders and behavioral impulses, transfer aggressive social cognitive biases, increase internalization and externalization problems, and form negative cognition of the real world. Therefore, harsh parenting can positively predict children’s normative beliefs about aggression.

At the same time, normative beliefs about aggression are an important predictor of aggressive behavior. Based on the information processing model of aggression development, normative beliefs about aggression can influence children’s behavioral choice, and help children manage aggressive behavior by a series of internal or external standards [17,20]. Social Information Processing (SIP) also points out that normative beliefs about aggression will adjust whether individuals exhibit aggressive behavior and control the frequency of aggressive behavior from a cognitive perspective [21]. Normative beliefs about aggression is positively correlated with actual aggressive behavior [22]. Children who agree more with indirect aggression do have higher indirect aggression in self-reports [23]. It also has a lasting impact. In other words, children may form higher normative beliefs about aggression by observing and learning their parents’ harsh parenting style, and these beliefs will further affect their aggressive behavior, revealing that is an important factor in children’s cognition. Therefore, this study hypothesizes that normative beliefs about aggression plays a mediating role between harsh parenting and the aggressive behavior of children.

### 1.2. The Moderating Role of Emotion Regulation Self-efficacy

General aggression model and social information processing model mainly understand aggression from the perspective of cognitive processing, however it is also worth noting that people’s emotions, cognition, and behavior are inseparable, and emotion has been proven to be an important information source for controlling and regulating behavioral responses [24]. Lemerise integrated the emotional process, including emotional regulation into the social information processing model and proposed an integrated model of emotional processes and cognition. The model holds that emotional regulation will affect the cognitive processing of social information, and finally affect behavioral decision making in social situations [25]. In the development of aggressive behavior, the role of emotion and cognition runs through all the time [26]. Researchers believe that children’s emotional tendency is one of the unique factors affecting their aggressive behavior in the dual-mode social information processing model–the latest explanation of differences in children’s aggressive behavior, which will affect their aggressive behavior through interaction with other factors [27]. Therefore, it is necessary to explore the development of children’s aggressive behavior from the integrative view of emotion, cognition, and behavior.

Regulatory emotional self-efficacy is an individual’s confidence in whether one can effectively regulate one’s emotional state, which can be divided into two categories: Self-efficacy in expressing positive emotions and self-efficacy in managing negative emotions. This factor is the potential influencing factor of individual internalization and externalization problems at an early stage [28]. Caprara et al. points out that people’s differences in managing their emotions lies not only in their ability to effectively use emotional regulation strategies and skills, but also in their belief to regulate their emotions. Individuals who are more confident in effectively regulating their emotional state will reduce the influence of stressful situations on themselves [29]. Teenagers with more externalization and internalization symptoms always have the experience of failing to control bad moods and maintain a stable emotional state at an early stage, which also means that their regulatory emotional self-efficacy is low [30]. When children’s regulatory emotional self-efficacy is low, they will not deliberately control their anger, nor pay attention to depression, and thus be more aggressive [31].

**Figure 1:**
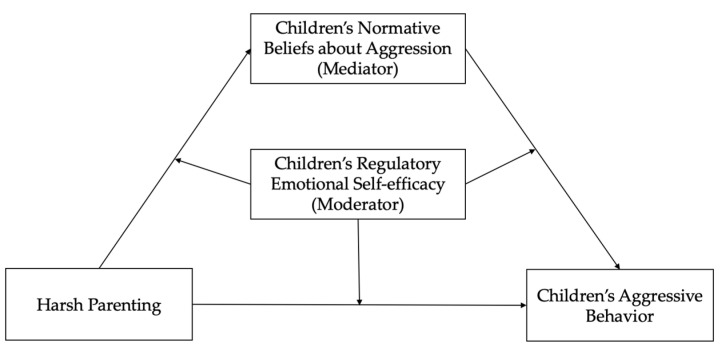
The proposed moderated mediation model.

The current research involves the mediating role of regulatory emotional self-efficacy between other factors and children’s aggressive behavior [32,33]. However, according to the dual-mode social information processing model of children’s aggressive behavior, children with a low emotional regulation ability indicate more behavioral problems, while children with a high emotional regulation ability have a lower risk of having behavior problems in the same situation [27], which means that emotion regulation self-efficacy may also play a moderating role in the development of children’s aggressive behaviors. Given this information, this study puts forward the hypothesis that children’s regulatory emotional self-efficacy would moderate the prediction model of harsh parenting and normative beliefs about aggression towards children’s aggressive behavior (H2). The whole model is shown in Figure 1. In addition, there are gender differences in the development of children’s aggressive behavior, as boys are usually more likely to show aggressive behavior than girls [34]. Therefore, in this study, we will control gender variables to explore whether the model has universal significance.

## 2. Materials and Methods

### 2.1. Participants

A cluster sampling method was used in this study, with 261 questionnaires distributed to students in grade 4, grade 5, and grade 6 in a primary school in Beijing. A total of 235 questionnaires (90.3%) were valid after screening according to the following exclusion criteria: (1) Participants did not fill in questionnaires completely, (2) the polygraph question in participants’ questionnaires were wrong, and (3) participants’ parents have both passed away. These 235 students included 126 boys (53.6%) and 109 girls (46.4%). The proportion of fourth-grade students, fifth-grade students, and sixth-grade students in the overall sample is 34.0%, 37.4%, and 28.5%, respectively.

### 2.2. Measures

#### 2.2.1. Harsh Parenting

The Harsh Parenting Scale adapted by Miao Tian et al. [35] on the basis of Wang’s research was used [36]. The scale consisted of 8 items, including 4 identical items for the father and mother. The children responded on a five-point scale ranging from 1 (never) to 5 (always). Children scored by recalling their parents’ behaviors, and the higher the score, the higher the children perceived the harsh parenting of their parents. The Cronbach alpha of this scale in this study is 0.844.

#### 2.2.2. Normative Beliefs about Aggression

In this study, the Normative Beliefs about Aggression Scale compiled by Huesman and Guerra was used. The Chinese version of the scale has good reliability and validity [37]. There are 20 items in total, including 12 items of revenge aggressive belief and 8 items of general aggressive belief. The items were rated on a four-point scale ranging from 1 (very wrong) to 4 (very correct), of which 17–20 were entitled reverse scores. Each topic had to be evaluated according to their own situation. The higher the score, the higher the recognition degree of aggressive behavior and the higher the normative beliefs about aggression. In this study, the Cronbach alpha of this scale is 0.892.

#### 2.2.3. Aggressive Behavior

The revised Buss–Warren Aggression Questionnaire (BWAQ) is one of the most widely-used assessment tools [38]. The instrument includes 34 items, which are divided into five dimensions, namely physical aggression, verbal aggression, indirect aggression, hostility, and anger. The scores range from 1 (not like me at all) to 5 (very like me), among which question 19 adopts the reverse scoring method, and the higher the score, the more aggressive behaviors are expressed. In this study, Cronbach’s alpha is 0.867.

#### 2.2.4. Regulatory Emotional Self-efficacy

On the basis of the Scale of Regulatory Emotional Self-efficacy (SRESE) compiled by Caprara [29], we revised the "Pupils’ Regulatory Emotional Self-efficacy Scale" according to Zhang’s method [39], as the scale has better applicability to primary school students. There are 24 items, which are divided into four dimensions: expressing happiness, managing anger, expressing pride, and expressing depression. The score ranges from 1 (not like me at all) to 5 (very like me). The higher the score, the stronger the self-efficacy of emotion regulation. In this study, Cronbach’s alpha is 0.908.

### 2.3. Procedure and Analytic Strategies

Before the study, the class teachers handed over the informed consent statement to the students and their guardians to sign, so as to ensure that the participants were clear about the purpose of the study and their rights and obligations. Then, the class teachers and research assistants used consistent instructions and required students to complete answers according to the instructions. All questionnaires were filled out by students themselves, and then the researchers took them back and logged data uniformly. After that, the data were analyzed by SPSS 26.0, and the specific analysis steps were as follows: (1) The common method bias was checked by Harman’s single-factor test. There were 23 factors with characteristics greater than 1, and the variation explained by the first factor was 17.6% (less than 40% of the critical value), indicating that there was no serious common method bias in this study; (2) descriptive statistics and the Pearson correlation analysis of variables were carried out; and (3) the PROCESS Macro for SPSS made by Hayes et al. [40] was used for a conditional process analysis. Model 59 was selected, the sample size was 5000, and the nonparametric percentile Bootstrap method for deviation correction was selected. The confidence level of the confidence interval was 95%, and the grouping criteria were that mean or addition or subtraction of the mean of one standard deviation.

### 2.4. Ethics Statement

This study was approved by the Ethics Committee of the Department of Psychology, School of Humanities and Social Sciences, Beijing Forestry University (no.: 2021-R15, date: 8 June 2021).

## 3. Results

### 3.1. Preliminary Analysis

Table 1 shows the means, standard deviations, and Pearson correlations of the study variables. The results revealed that harsh parenting was positively correlated with children’s aggressive behavior (*r* = 0.37, *p* < 0.01), children’s normative beliefs about aggression was positively correlated with gender (*r* = 0.20, *p* < 0.01), harsh parenting (*r* = 0.59, *p* < 0.01), and children’s aggressive behavior (*r* = 0.47, *p* < 0.01). In addition, children’s regulatory emotional self-efficacy was negatively correlated with harsh parenting (*r* = −0.13, *p* < 0.05), children’s aggressive behavior (*r* = −0.31, *p* < 0.01), and normative beliefs about aggression (*r* = −0.34, *p* < 0.01).

### 3.2. Hypotheses Testing

Firstly, on the basis of correlation analysis, we used PROCESS Macro for SPSS [40] to test the hypotheses after controlling for the impact of gender. As shown in Table 2, harsh parenting has a significant positive predictive effect on children’s normative beliefs about aggression (*β =* 0.50, *p* < 0.001) and aggressive behavior (*β =* 1.42, *p* < 0.001), and children’s normative beliefs about aggression has a significant positive predictive effect on aggressive behavior (*β* = 0.35, *p* < 0.001). This indicated that children’s normative beliefs about aggression plays a mediating role in the relationship between harsh parenting and children’s aggressive behavior, thus supporting hypothesis 1.

**Figure 2:**
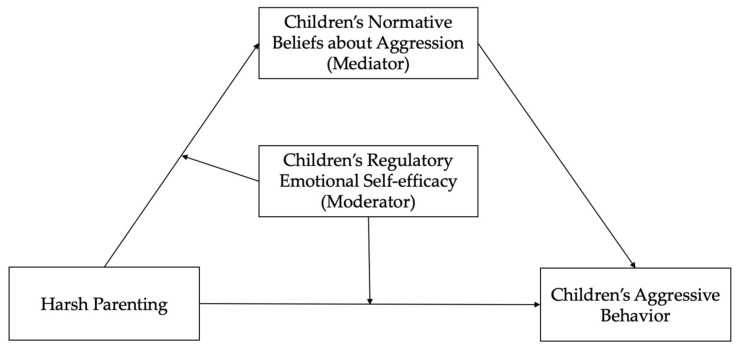
The modified moderated mediation model.

Besides, the interaction term between harsh parenting and children’s regulatory emotional self-efficacy can significantly negatively predict children’s normative beliefs about aggression (*β* = −0.01, *p* < 0.05) and aggressive behavior (*β* = −0.03, *p* < 0.001), which shows that the influence of harsh parenting on children’s normative beliefs about aggression and aggressive behavior is moderated by children’s regulatory emotional self-efficacy. However, the interaction term between children’s normative beliefs about aggression and regulatory emotional self-efficacy cannot significantly predict children’s aggressive behavior (*p* > 0.05), which shows that children’s regulatory emotional self-efficacy cannot play a moderating role in the influence of children’s normative beliefs about aggression on aggressive behavior. In general, the direct effect of harsh parenting on children’s aggressive behavior and the first half of the mediating effect of children’s normative beliefs about aggression in the hypothesis model (see Figure 1) are both moderated by children’s regulatory emotional self-efficacy, however emotion-regulated self-efficacy cannot play a moderating role in the second half of the mediating effect. The modified model is shown in Figure 2.

Secondly, with M ± SD as the standard, we differentiated the low, middle, and high levels of children’s regulatory emotional self-efficacy, and the direct effect of harsh parenting on children’s aggressive behavior and the indirect effect on children’s normative beliefs about aggression were analyzed at different levels. As shown in Table 3, at three levels of children’s regulatory emotional self-efficacy, harsh parenting showed to have a significant impact on children’s aggressive behavior (95% Bootstrap CI = [1.56,2.39], [1.14,1.70], [0.51,1.22]). Besides, the indirect effect of harsh parenting on children’s aggressive behavior through children’s normative beliefs about aggression is significant at middle and high levels of children’s regulatory emotional self-efficacy (95% Bootstrap CI = [0.06,0.32], [0.03,0.29]), however it was not significant at a low level (95% Bootstrap CI = [−0.10,0.40]). This indicates that children’s regulatory emotional self-efficacy can not only moderate the direct effect of harsh parenting on children’s aggressive behavior, but also moderate the indirect effect of children’s normative beliefs about aggression in the relationship between harsh parenting and children’s aggressive behavior. Therefore, hypothesis 2 was supported.

**Figure 3:**
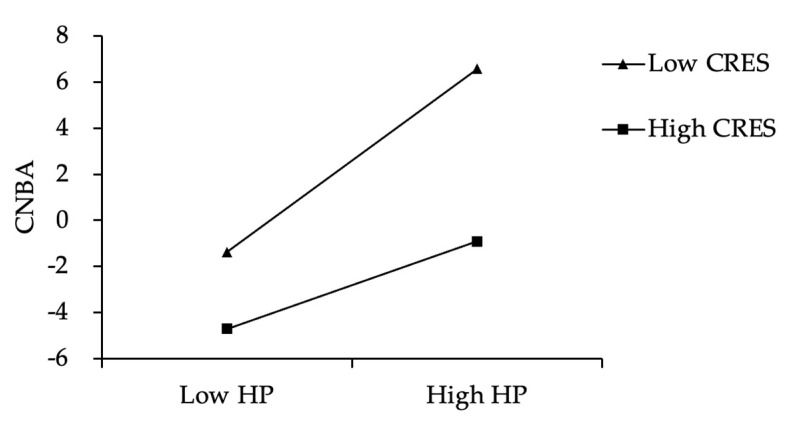
CRES moderates the effect of HP on CNBA. Note: HP = harsh parenting; CNBA = children’s normative beliefs about aggression; CRES = children’s regulatory emotional self-efficacy.

**Figure 4:**
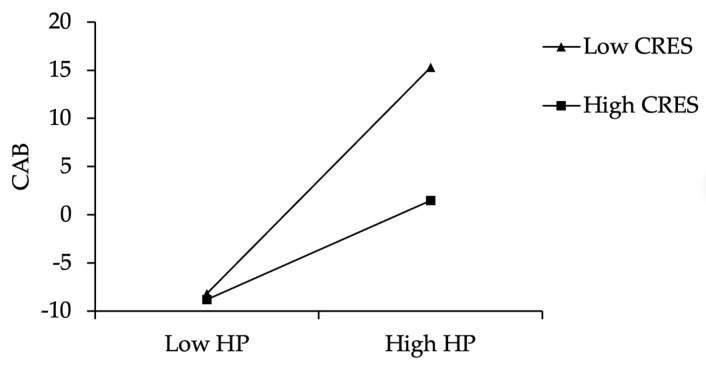
CRES moderates the effect of HP on CNBA. Note: HP = harsh parenting; CNBA = children’s normative beliefs about aggression; CRES = children’s regulatory emotional self-efficacy.

Furthermore, we used simple slope analysis to better explain the moderating effect of children’s regulatory emotional self-efficacy. As shown in Figure 3, for children with lower regulatory emotional self-efficacy, harsh parenting can significantly predict their normative beliefs about aggression (*simple slope* = 0.67, *t* = 5.03, *p* < 0.001), but for children with higher regulatory emotional self-efficacy, harsh parenting has relatively little predictive effect on their normative beliefs about aggression (*simple slope* = 0.32, *t* = 2.66, *p* <0.01). In addition, as shown in Figure 4, for children with lower regulatory emotional self-efficacy, harsh parenting can significantly predict their aggressive behavior (*simple slope* = 1.98, *t* = 9.36, *p* < 0.001), but for children with higher regulatory emotional self-efficacy, harsh parenting has relatively little predictive effect on their aggressive behavior (*simple slope* = 0.86, *t* = 4.81, *p* < 0.001). In summary, with the improvement of children’s regulatory emotional self-efficacy, the predictive effect of harsh parenting on their normative beliefs about aggression and aggressive behavior will both gradually weaken.

## 4. Discussion

Based on the integrated model of emotional processes and cognition [25] and the dual-mode social information processing model [27], this study constructs a moderated mediation model with normative beliefs about aggression as mediators and regulatory emotional self-efficacy as a moderator. After clarifying the mechanism between harsh parenting and children’s aggressive behavior (the mediating role of normative beliefs about aggression), it further points out the conditions under which the impact of harsh parenting on children’s aggressive behavior can be alleviated (the moderating role of regulatory emotional self-efficacy). The results have theoretical and practical significance for combing the role of emotion and cognition in the development of children’s aggressive behavior and reducing children’s aggressive behavior.

### 4.1. The Mediating Role of Normative Beliefs about Aggression

The results of this study show that harsh parenting can positively predict children’s aggressive behavior through the mediating role of normative beliefs about aggression, which is consistent with hypothesis 1 (H1). Specifically, a harsh parenting environment will enhance children’s acceptance of violence or aggression, and children are more likely to attribute other people’s behaviors to aggressive intentions, and think that revenge and aggressive behaviors are more in line with social norms, thus having a higher level of normative beliefs about aggression. Therefore, when they encounter conflict situations, they prefer to use aggressive ways to solve problems, rather than adopting a more rational or peaceful way.

First of all, this result makes clear the influence of harsh parenting on children, that is, harsh parenting can significantly positively predict children’s aggressive behavior, which is consistent with the results of previous studies [13,14]. There are various factors leading to this phenomenon. From the physiological factors, children in harsh parenting environment will have a lower RSA (resting respiratory sinus arrhythmia is a biological indicator of stress sensitivity) level. Individuals with a lower level will be more sensitive to the environment. Long-term family stress may inhibit the development of children’s RSA, which increases the possibility of children’s aggressive behavior [41]. This study explores the role of cognitive processing factors and emotional factors in this mechanism, and the research group is children from China. It not only expands the research group, but also explains its role across regions and ages.

Secondly, this study focuses on the perspective of cognitive processing, further clarify the mechanism of the influence of harsh parenting on children’s aggressive behavior. That is to say, the normative beliefs about aggression will play a mediating role in this mechanism, which supports the social information processing model. This model points out that children have to go through six cognitive processes before reaching aggression. The details are as follows: Encoding of cues, interpretation of cues, clarification of goals, response access or construction, response decision, and behavior enactment [21]. Aggressive children will have cognitive defects in these processes. This is the reason why they are more likely to produce hostile attribution and aggressive behavior. The mediating role of normative beliefs about aggression further clarifies where the cognitive defect is reflected. At the same time, it also supports the basic viewpoint of a general aggression model, that is, cognitive factors are important mediating variables between the environment and individual characteristics and aggression [42]. In addition, an environment of violence conflict tends to improve normative beliefs about aggression and make them acquire aggressive behavior as a way to manage problems [43]. When individuals are in a high-pressure psychological environment for a long time, their hostility, insecurity, and negative emotional experience will be stronger, which will easily lead to cognitive bias on social information and eventually induce a series of problem behaviors, including aggressive behaviors [44,45].

In a word, harsh parenting can be regarded as a violent and high-pressure environment for children’s growth. As a clue to children’s coding, a harsh parenting environment will easily make children become accustomed to violence. They are indifferent to the harmful consequences of violence, and think that violence is universal and inevitable, and recognize the problem-solving methods of violence, which will lead to the improvement of their normative beliefs about aggression, thus being more likely to show aggressiveness under certain situational clues. The results of this study enlighten us that parents should construct a good family environment, use a more active parenting style, and avoid harsh parenting. At the same time, in the prevention and intervention of children’s aggressive behavior in the future, we should also pay attention to the role of cognitive systems, such as normative beliefs about aggression. Good publicity and education can help children decrease their recognition of aggressive behavior and reduce the occurrence of aggressive behavior.

### 4.2. The Moderating Role of Emotion Regulation Self-efficacy

An important finding of this study is the moderating role of emotion regulation self-efficacy in the relationship between harsh parenting and children’s aggressive behavior. The results find that children’s emotion regulation self-efficacy can not only regulate the relationship between harsh parenting and aggressive behavior, but can also play a moderating role in the first half of the intermediary chain of "harsh parenting → normative beliefs about aggression → aggressive behavior", which is partially consistent with hypothesis 2 (H2). This reflects the role of the emotional process in the development of children’s aggressive behavior.

This conclusion supports the integrated model of emotional processes and cognition [25], which outlines that the emotional process can play a role in every stage of children’s social information processing. For example, in the stage of cues encoding and interpretation, the individual emotional process will affect the encoding and interpretation of cues, and these cues may also trigger individual emotional changes. In the stage of goal clarification, emotions can provide energy for goal selection or classification, and goal selection or maintenance can adjust emotion or mood. In the stage of response access or construction and response decision, children’s emotional experience will affect the reaction evaluation and decision making, and specific reaction evaluation and decision making will in turn adjust emotions. In the stage of response execution, emotional processes including emotional experience and emotional regulation can affect children’s final behavior and response measures. The model has been proven in the research of children, adolescents, and adult groups [46,47,48].

Based on the integrated model of emotional processes and cognition, this study further found that for children with high regulatory emotional self-efficacy, harsh parenting has a weak predictive effect of harsh parenting on their normative beliefs about aggression and aggressive behavior. The results not only show that there are individual differences in the cognitive mechanism of children’s aggressive behavior (the mediating effect of aggressive normative beliefs), but also show that regulatory emotional self-efficacy is a protective factor to avoid the negative impact of harsh parenting on children’s aggressive behavior, which is consistent with previous theories and research results [27,49]. Specifically, children who believe that they can effectively regulate their emotional state will have a stronger ability in controlling their emotions, so they can better face the pressure of the environment [50]. In this state, they can adopt positive coping strategies to reduce the negative impact of harsh parenting and avoid forming recognition of aggressive behavior, and ultimately reduce the frequency of aggression. Therefore, parents and schools can pay attention to the cultivation of children’s regulatory emotional self-efficacy, so as to prevent and intervene with children’s aggressive behavior.

Remarkably, in this study, children’s regulatory emotional self-efficacy cannot play a moderating role in the second half of the intermediary chain of “harsh parenting → normative beliefs about aggression → aggressive behavior”, that is, children’s regulatory emotional self-efficacy cannot regulate the relationship between children’s normative beliefs about aggression and aggressive behavior. When children have recognized aggressive behavior in cognition, children’s confidence in whether they can effectively regulate their emotional state can no longer affect the decision making and implementation of aggressive behavior. This shows that the development of children’s aggressive behavior is characterized by stages [51], and emotional processes play different roles in different stages of social information processing [25]. Therefore, it is necessary to prevent and intervene with children’s aggressive behavior as soon as possible.

### 4.3. Limitations and Further Research

Although this study explores the relationship between harsh parenting and children’s aggressive behavior from the comprehensive perspectives of emotion, cognition, and behavior, there are some limitations: (1) This study adopts cross-sectional approach, so it is impossible to determine whether the research results will change with time, and it is difficult to determine the causal link. Later research can adopt follow-up research and collect data according to certain time intervals to further explore the relationship between harsh parenting and children’s aggressive behavior. (2) This study does not distinguish the aggressive behavior of children, for example, active aggression and reactive aggression. Previous studies on social information processing models show that reactive aggression is mainly related to social cognitive bias at an early stage (coding of situation cues and attribution of hostile intention), while active aggression is mainly related to social cognitive bias in the later stage (decision making and execution of aggressive behavior) [52]. This shows that different types of aggression may be affected by different cognitive processing stages, and later studies can distinguish them, and further explore the relationship between children’s regulatory emotional self-efficacy or other emotional process factors in harsh parenting and children’s aggressive behavior development.

## 5. Conclusions

(1) Harsh parenting had a significant positive predictive effect on children’s aggressive behavior.

(2) Normative beliefs about the aggression of children mediated the relationship between harsh parenting and children’s aggressive behavior.

(3) Regulatory emotional self-efficacy had moderating effects both in the direct predictive effect of harsh parenting on children’s aggressive behavior and in the first half of the intermediary chain of “harsh parenting → normative beliefs about aggression → aggressive behavior”.

## Figures and Tables

**Table 1:** Means, standard deviations, and correlations among the variables.

Variables	M	SD	Gender	HP	CAB	CNBA	CRES
Gender	0.54	0.50	1.00				
HP	13.85	6.01	0.13	1.00			
CAB	32.78	9.42	0.11	0.37 **	1.00		
CNBA	59.64	16.67	0.20 **	0.59 **	0.47 **	1.00	
CRES	85.32	18.11	0.09	−0.13 *	−0.31 **	−0.34 **	1.00

Note: N = 235; Gender was dummy coded as 0 (= female) and 1 (= male); HP = harsh parenting, CAB = children’s aggressive behavior, CNBA = children’s normative beliefs about aggression, CRES = children’s regulatory emotional self-efficacy; SD = standard deviation; * *p* < 0.05, ** *p* < 0.01.

**Table 2:** Results of the hypotheses testing.

Regression Equation		Fitting Index			Coefficient Significance		
Outcome Variables	Predictor Variables	R	R^2^	F	β	95%CI	t
CNBA	Gender	0.50	0.25	19.43 ***	3.42	[1.27, 5.57]	3.13 ***
	HP				0.50	[0.31, 0.68]	5.41 ***
	CRES				−0.15	[−0.21, −0.09]	−4.95 ***
	HP × CRES				−0.01	[−0.02, −0.01]	−2.00 *
CAB	Gender	0.71	0.50	38.01 ***	0.73	[−2.47, 3.92]	0.45
	HP				1.42	[1.14, 1.70]	10.01 ***
	CNBA				0.35	[0.16, 0.54]	3.67 ***
	CRES				−0.20	[−0.29, −0.11]	−4.28 ***
	HP × CRES				−0.03	[−0.46, −0.02]	−4.11 ***
	CNBA × CRES				0.01	[−2.47, 3.92]	0.45

Note: N = 235; Gender was dummy coded as 0 (= female) and 1 (= male); HP = harsh parenting, CAB = children’s aggressive behavior, CNBA = children’s normative beliefs about aggression, CRES = children’s regulatory emotional self-efficacy; SD = standard deviation; * *p* < 0.05, *** *p* < 0.001.

**Table 3:** Results of the influence of HP on CAB at different levels of CRES.

Moderator	Direct effect of CAB			Indirect effect of CNBA		
	Effect	Boot SE	95% Bootstrap CI	Effect	Boot SE	95% Bootstrap CI
Low-CRES	1.98	0.21	[1.56,2.39]	0.25	0.17	[−0.10,0.40]
Middle-CRES	1.42	0.14	[1.14,1.70]	0.19	0.17	[0.06,0.32]
High-CRES	0.86	0.18	[0.51,1.22]	0.12	0.14	[0.03,0.29]

Note: HP = harsh parenting, CAB = children’s aggressive behavior, CNBA = children’s normative beliefs about aggression, CRES = children’s regulatory emotional self-efficacy; Low-CRES = M − SD, Middle-CRES = M, High-CRES = M + SD; SD = standard deviation.

## Data Availability

No publicly archived datasets analyzed or generated were used in this study.
